# Evaluating the association of common *PBX1 *variants with type 2 diabetes

**DOI:** 10.1186/1471-2350-9-14

**Published:** 2008-02-29

**Authors:** Konsta Duesing, Guillaume Charpentier, Michel Marre, Jean Tichet, Serge Hercberg, Beverley Balkau, Philippe Froguel, Fernando Gibson

**Affiliations:** 1 Genomic Medicine, Imperial College London, Hammersmith Campus, Du Cane Rd, London W12 0NN, UK; 2 Endocrinology-Diabetology Unit, Corbeil Hospital, Corbeil, France; 3 Endocrinology-Diabetology, Bichat Hospital, Paris, France; 4 INSERM U695, Paris, France; 5 Institut Régional Pour la Santé, Tours, France; 6 U557 Inserm/U1125 Inra/Cnam/University Paris 13, CRNH IdF, F-93017 Bobigny, France; 7 INSERM U780-IFR69, Villejuif, France; 8 Paris Univ-Sud, Orsay, France; 9 CNRS 8090, Institut de Biologie de Lille, Institut Pasteur, Lille, France

## Abstract

**Background:**

*PBX1 *is a biological candidate gene for type 2 diabetes at the 1q21-q24 susceptibility locus. The aim of this study was to evaluate the association of common *PBX1 *variants with type 2 diabetes in French Caucasian subjects.

**Methods:**

Employing a case-control design, we genotyped 39 SNPs spanning the *PBX1 *locus in 3,093 subjects to test for association with type 2 diabetes.

**Results:**

Several *PBX1 *SNPs, including the G21S coding SNP rs2275558, were nominally associated with type 2 diabetes but the strongest result was obtained with the intron 2 SNP rs2792248 (P = 0.004, OR 1.20 [95% CI 1.06–1.37]). The SNPSpD multiple testing correction method gave a significance threshold of P = 0.002 for the 39 SNPs genotyped, indicating that the rs2792248 association did not survive multiple testing adjustment. SNP rs2792248 did not show evidence of association with the French 1q linkage signal (P = 0.31; weighted NPL score 2.16). None of the *PBX1 *SNPs nominally associated with type 2 diabetes were associated with a range of quantitative metabolic traits in the normoglycemic control subjects

**Conclusion:**

The available data does not support a major influence of common *PBX1 *variants on type 2 diabetes susceptibility or quantitative metabolic traits. In order to make progress in identifying the elusive susceptibility variants in the 1q region it will be necessary to carry out further large association studies, meta-analyses of existing data from individual studies, and deep resequencing of the 1q region.

## Background

*PBX1 *is a strong biological candidate gene at the chromosome 1q21-q24 susceptibility locus [1]. It is a member of the TALE (three amino acid loop) class of homeodomain factors which regulate developmental gene expression in heteromeric complexes with other transcription factors [2]. Pbx1 null mice exhibit severe defects in pancreatic exocrine and endocrine cell differentiation and die at E15-16, while Pbx1^+/- ^mice have pancreatic islet malformation, impaired glucose tolerance and hypoinsulinemia [3]. *In vitro *studies showed that Pbx1 binds cooperatively with Pdx1 to the regulatory elements of target pancreatic genes, such as somatostatin and elastase [4-6]. Pdx1^-/- ^mice expressing a Pbx1-interaction defective transgene contain all the various pancreatic cell types, but have markedly smaller pancreata and islets than Pdx1^-/- ^mice expressing the wild-type Pdx1 transgene [7]. This suggested that Pbx1:Pdx1 complexes are essential for normal proliferation after pancreatic cell type differentiation. In addition, compound heterozygote Pbx1^+/- ^Pdx1^+/- ^mice exhibit an age-dependent diabetes phenotype [7]. These findings demonstrated that, at least in mice, Pbx1 plays a key role in pancreatic development and that Pbx1 is an important cofactor of Pdx1, a 'master regulator' of pancreatic development and function [8]. Wang et al. [9] reported the results of a small case-control study (192 diabetic cases and 192 normoglycemic controls) in which 20 *PBX1 *variants were evaluated for association with type 2 diabetes in Utah Caucasians. Three variants were nominally associated (P < 0.05) with type 2 diabetes, as were haplotypes involving intron 2 variants. In the present study, we set out to perform a large association study of common *PBX1 *variation and type 2 diabetes in French Caucasians.

## Methods

### Case-control subjects

All subjects were of French Caucasian ancestry. Individuals identified by Sladek et al. [10] to lie outside the HapMap CEU ancestry cluster were excluded from the study. Type 2 diabetic case subjects were known diabetic patients. Normoglycemic control subjects were selected to have a fasting blood glucose concentration < 6.1 mM [11]. Case subjects were composed of: (i) 372 probands from diabetic families, recruited in Lille; and (ii) 1083 patients with a family history of T2D recruited at the Corbeil-Essonne Hospital. Control subjects were composed of: (i) 372 normoglycemic parents from T2D families; (ii) 524 subjects from the SUVIMAX prospective population-based cohort study [12]; and (iii) 742 subjects selected from the DESIR cohort, a large prospective study of insulin resistance in French subjects [13]. In total, the case-control cohort comprised 1,455 type 2 diabetic subjects (age, 60 ± 12 years; BMI, 29.0 ± 6.0 kg/m^2^; male/female, 56:44%) and 1,638 normoglycemic subjects (age, 54 ± 13 years; BMI, 24.1 ± 3.3 kg/m^2^; male/female, 43:57%). At α = 0.05, this sample size provides 94% power to detect a susceptibility allele with a frequency of 0.25, assuming a disease prevalence of 0.1 and a heterozygote relative risk of 1.2 (multiplicative model) [14]. Informed consent was obtained from all subjects and the study was approved by the local ethics committees.

### PBX1 resequencing

The genomic target sequences for *PBX1 *resequencing consisted of each of the 9 exons (NCBI RefSeq NM_002585) together with ~500 bp flanking intronic sequence, 500 bp of the 3' UTR and 1 kb of the 5' UTR. We sequenced these regions in 24 unrelated probands taken from families with the strongest evidence of linkage at 1q (NPL score ≥ 0.816) [15]. PCR reactions (primer sequences are available on request) were performed with 100 ng human genomic DNA and AmpliTaq Gold polymerase (Applied Biosystems, USA) using standard PCR conditions in a total volume of 25 μl. Bi-directional sequencing reactions were performed with the BigDye Terminator v.3.1 cycle sequencing kit (Applied Biosystems, USA) and electrophoresed on the Applied Biosystems 3700 Genetic Analyzer according to the manufacturer's instructions. Sequence alignment and analysis were carried out with the Phred/Phrap/Consed system [16,17].

### *PBX1 *SNP identification and selection

This study was initiated well before completion of the HapMap project [18], and we employed a combination SNP selection strategy involving: i) resequencing; ii) the identification of preliminary association signals (P < 0.05) in the French case-control samples (n = 600) of the International Type 2 Diabetes 1q Consortium dataset; and iii) the extraction of SNPs from dbSNP. Resequencing identified four common (MAF ≥ 5%) SNPs. The 1q Consortium data highlighted six interim association signals at the *PBX1 *locus, including the G21S variant and SNPs in the 5' upstream region and the large 2nd intron (229 kb). To provide additional genomic and functional coverage in these regions, we identified common validated SNPs in: ~35 kb of the 5' upstream region at a density of 1.5 SNPs/kb; and conserved noncoding regions (CNRs) in introns 1 and 2. CNRs were defined as ≥ 80% human-mouse identity across a 100 bp window in the VISTA database [19]. In total, 39 SNPs at the *PBX1 *locus were tested for association with type 2 diabetes.

### SNP genotyping

Genotyping was performed with the Sequenom MassARRAY system [20]. SNP genotype frequencies were tested for accordance with Hardy-Weinberg equilibrium using chi-squared analysis. Quality control criteria: SNPs with a call rate < 90%, a MAF < 5%, a Hardy-Weinberg P < 0.05, or that exhibited poorly defined genotype clusters were disqualified from association analysis.

### Statistical analyses

To test the association of *PBX1 *SNPs with type 2 diabetes, chi-squared analysis of allelic and genotype counts was performed. To address the multiple testing problem, the SNPSpD method [21] was employed. Pairwise SNP LD values were calculated and plotted with Haploview [22]. Quantitative metabolic phenotypes measured in the 1,638 normoglycemic subjects that comprised the control cohort were log transformed and adjusted for age, sex and BMI, as appropriate. SNPs were tested for association with adjusted quantitative traits using SPSS 14.0 with the ANOVA test under a codominant model. The program GIST [23] was used to assess the contribution of SNPs to the 1q linkage signal previously obtained with the 56 "strict lean" French families [15]. Meta-analysis was performed with the Mantel-Haenszel [24] method estimating the common odds ratio for combined allele counts using SPSS 14; results are reported for the two-sided, asymptotically distributed test.

## Results

The *PBX1 *gene locus extends for 287 kb (162,795,561..163,082,934 bp NCBI36) on chromosome 1q23 and consists of 9 exons. We tested 39 SNPs spanning the *PBX1 *locus for association with type 2 diabetes in 3,093 French subjects. Genotype call rates exceeded 90% overall. The overlap between samples in the present study and that of the 1q Consortium allowed us to determine the genotype concordance rates for the six SNPs genotyped in common between the two datasets. Concordance rates were all greater than 98% (Table S1, Additional file 1). The allelic and genotype count data for all genotyped SNPs is presented in Tables S2 and S3, respectively (additional file 1). Several *PBX1 *SNPs, including the G21S variant, were modestly associated with type 2 diabetes (Table 1), but the strongest result was obtained with the intron 2 SNP rs2792248 (P = 0.004, OR 1.21 [95% CI 1.06–1.39]), which had shown preliminary association with type 2 diabetes in the 1q Consortium dataset. However, the SNPSpD multiple testing correction method [21] gave a significance threshold of P = 0.002 for the 39 SNPs genotyped (equivalent to 21 independent tests), indicating that the rs2792248 result did not survive multiple testing adjustment. There were no significant differences in SNP allele frequencies between males and females (data not shown).

**Table 1:** *PBX1 *SNPs nominally associated with type 2 diabetes in French Caucasians.

**SNP**	**Allele^b^**	**Chr Position (NCBI36)**	**Gene Region**	**n subjects**		**Allele 1 (%)**	**Allele 2 (%)**	**P**	**OR **(95% CI)
rs10918027	A/G	162765790	5' upstream	T2D	1414	2128 (75)	700 (25)	0.040	1.14 (1.01–1.28)
				NG	1567	2429 (78)	705 (22)		
rs1338625	A/C	162780889	5' upstream	T2D	1420	2107 (74)	733 (26)	0.039	1.13 (1.01–1.27)
				NG	1576	2411 (76)	741 (24)		
rs6662567	C/T	162783473	5' upstream	T2D	1433	2140 (75)	726 (25)	0.040	1.13 (1.01–1.27)
				NG	1574	2422 (77)	726 (23)		
rs6426870	T/C	162790577	5' upstream	T2D	1396	2073 (74)	719 (26)	0.014	1.16 (1.03–1.31)
				NG	1542	2375 (77)	709 (23)		
rs2275558 (G21S) ^a^	G/A	162795744	Exon 1	T2D	1427	2193 (77)	661 (23)	0.013	1.17 (1.03–1.32)
				NG	1626	2584 (79)	668 (21)		
rs2792248 ^a^	A/G	162891886	Intron 2	T2D	1178	1814 (77)	542 (23)	0.004	1.21 (1.06–1.39)
				NG	1442	2121 (74)	763 (26)		

^a ^SNPs that showed evidence of association with T2D (P < 0.05) in the French samples of the 1q Consortium; ^b ^SNP alleles are shown as major/minor.

**Figure 1:**
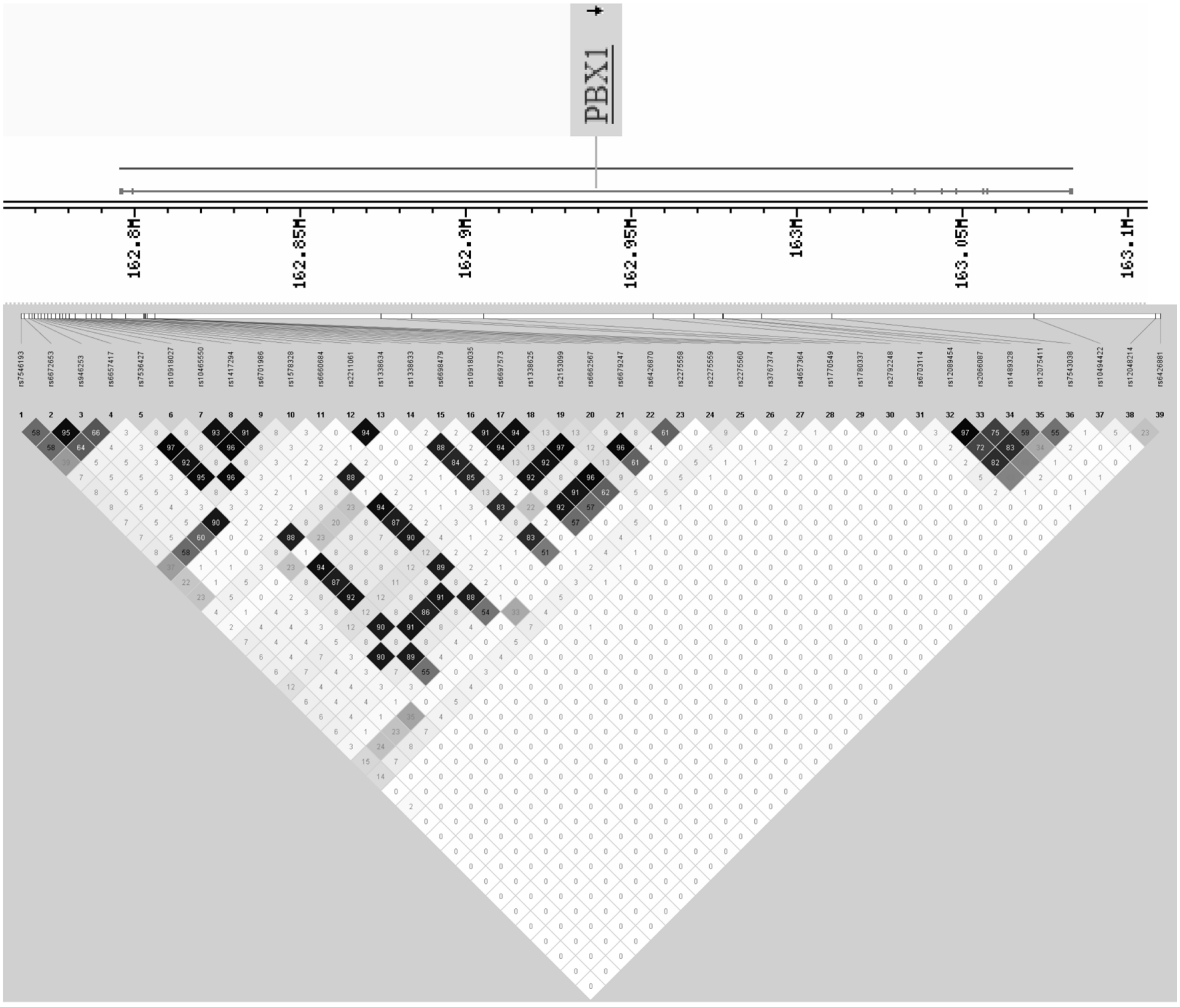
**Pattern of linkage disequilibrium across the *PBX1 *locus**. Haploview plots of pairwise SNP LD values (r^2^) calculated from the pooled case-control genotype data.

There was weak LD between the genotyped SNPs (Fig. 1) and thus we did not carry out haplotype analysis. In particular, SNP rs2792248 was not in LD with any other genotyped markers. SNP rs2792248 did not show evidence of contributing to the French 1q linkage signal (P = 0.31; weighted NPL score 2.16). Interestingly, we did obtain suggestive evidence under the additive model (P = 0.03; weighted NPL score 3.87) that the minor allele of the G21S variant was associated with the 1q linkage signal. Finally, none of the *PBX1 *SNPs nominally associated with type 2 diabetes were associated with a range of metabolic quantitative traits in the normoglycemic control subjects (Table S4, Additional file 1).

The availability of data from type 2 diabetes genome-wide association studies (GWASs) prompted us to carry out a meta-analysis of *PBX1 *variants genotyped in common between the present study and the GWASs. However, only three SNPs analysed here were successfully typed in the two GWASs that published summary statistics (the DGI [25] and WTCCC [26] studies). The nominally significant associations of the upstream variants rs1338625 and rs6426870 were not supported by the combined analysis (P = 0.62, OR 0.98 [95% CI 0.92–1.05]; P = 0.82, OR 0.99 [95% CI 0.93–1.06], respectively); while the negative result for rs4657364 was confirmed (P = 0.45, OR 1.03 [95% CI 0.95–1.12]) (Tables S5-7, Additional file 1).

## Discussion

We have examined a number of common SNPs spanning the *PBX1 *locus for association with type 2 diabetes in the French population and obtained several nominal association signals, of which the strongest was the intron 2 SNP rs2792248. Despite the finding that this association disappeared upon correction for multiple testing, this finding substantiated an association previously observed in the small French sample set (n = 600) available to the 1q Consortium. For this reason, it is possible that this is a bona fide, albeit minor, association signal that was overwhelmed by the sheer number of SNPs tested. Nevertheless, it appears unlikely from the data presented here (and elsewhere – see below) that common *PBX1 *variants have a major influence on susceptibility to type 2 diabetes. Before formally rejecting *PBX1 *as a type 2 diabetes susceptibility gene, however, we should make the point that *PBX1 *is a large gene and the current study does not provide comprehensive coverage of common variation at the *PBX1 *locus. According to HapMap data release #22, a total of 112 HapMap II tag SNPs are required to capture the common variation across the region covered in this study at r^2 ^≥ 0.8 and MAF ≥ 0.05. A comparison of this list with the SNPs genotyped in this study indicates that we typed a mere 18 tag SNPs (or proxies) plus 11 other SNPs, equating to ~25% of the common variation at *PBX1*. Thus, we are not able to rule out the possibility that type 2 diabetes susceptibility variants exist at the *PBX1 *locus. Nevertheless, our results are in general agreement with the type 2 diabetes GWASs, none of which found strong association signals at *PBX1 *or indeed anywhere within the 1q region [10,25-27].

The problem of identifying the genetic variants responsible for the 1q linkage signal(s) has proven to be refractory to progress, despite dense (1 SNP per 5 kb) region-wide genotyping by the International 1q Consortium, a number of individual candidate gene studies, as well as the genomewide association scans. This preponderance of negative results suggests that the various 1q linkages were generated by a combination of many different common and/or rare variants of small effect (OR 1.01–1.1) which reasonably large studies (i.e. samples numbering in the thousands) have only limited power to detect. If this is true, the way forward at 1q will be to identify the common, minor susceptibility variants with meta-analyses of genotype data from large individual studies; and to probe the contribution of rare variants by deep resequencing of the 1q region.

## Conclusion

The available data do not support a major influence of common *PBX1 *variants on type 2 diabetes susceptibility or quantitative metabolic traits. In order to make progress in identifying the elusive susceptibility variants in the 1q region it will be necessary to carry out further large association studies, meta-analyses of existing data from individual studies, and deep resequencing of the 1q region.

## Conflicting interests

The author(s) declare that they have no competing interests.

## Authors' contributions

KD participated in the design of the study, carried out the SNP genotyping, participated in the analysis of the genotype data and the drafting of the manuscript. GC contributed to the study design of the study. MM contributed to the study design. JT contributed to the study design. SH contributed to the study design. BB contributed to the study design. PF contributed to the study design and contributed to drafting the manuscript. FG contributed to the design of the study, the analysis of the genotype data and in drafting the manuscript. All authors read and approved the final manuscript.

## Pre-publication history

The pre-publication history for this paper can be accessed here:



## Supplementary Material

Additional file 1 Supplemental Tables longer than two sides of A4 in length and/or those that are not intended to appear in the body of the article. Click here for file
