# Supplementary material for: Evaluating the association of common PBX1 variants with type 2 diabetes
Source: BMC Med Genet. 2008 Feb 29;9:14. doi: 10.1186/1471-2350-9-14 (PMC2292156; doi:10.1186/1471-2350-9-14)
Supplement: Additional file 1 — Supplemental Tables longer than two sides of A4 in length and/or those that are not intended to appear in the body of the article. [file 1471-2350-9-14-S1.doc]

Table S1. The genotype concordance rates for the SNPs and samples genotyped in common in the present study and the 1q Consortium dataset.

| SNP | n subjects | Concordant genotypes | Concordance Rate (%) |
| --- | --- | --- | --- |
| rs946253 | 427 | 420 | 98.4 |
| rs1417294 | 468 | 462 | 98.7 |
| rs2275558 | 482 | 478 | 99.2 |
| rs2792248 | 419 | 411 | 98.1 |
| rs12089454 | 479 | 478 | 99.8 |
| rs12075411 | 479 | 478 | 99.8 |

Table S2. Association analysis of *PBX1* SNPs with type 2 diabetes in French Caucasians

| **SNP** | **SNP feature^a^** | **Allele^b^** | **Chr Position (NCBI36)** | **Gene Region** | **n subjects** | | **Allele 1 (%)** | **Allele 2 (%)** | **P** |
| --- | --- | --- | --- | --- | --- | --- | --- | --- | --- |
| rs7546193 |  | A/T | 162761029 | 5′ upstream | T2D | 1393 | 2134 (77) | 652 (23) | 0.368 |
|  |  |  |  |  | NG | 1568 | 2433 (78) | 703 (22) |  |
| rs6672653 |  | G/A | 162762036 | 5′ upstream | T2D | 1406 | 2376 (84) | 436 (16) | 0.714 |
|  |  |  |  |  | NG | 1609 | 2730 (85) | 488 (15) |  |
| rs946253 | 1qC | A/G | 162763235 | 5′ upstream | T2D | 1414 | 2381 (84) | 447 (16) | 0.620 |
|  |  |  |  |  | NG | 1585 | 2654 (84) | 516 (16) |  |
| rs6657417 |  | A/G | 162764152 | 5′ upstream | T2D | 1437 | 2543 (88) | 331 (12) | 0.530 |
|  |  |  |  |  | NG | 1581 | 2814 (89) | 348 (11) |  |
| rs7536427 |  | T/C | 162764848 | 5′ upstream | T2D | 1365 | 2128 (78) | 602 (22) | 0.539 |
|  |  |  |  |  | NG | 1569 | 2425 (77) | 713 (23) |  |
| rs10918027 |  | A/G | 162765790 | 5′ upstream | T2D | 1414 | 2128 (75) | 700 (25) | **0.040** |
|  |  |  |  |  | NG | 1567 | 2429 (78) | 705 (22) |  |
| rs10465550 |  | C/T | 162766657 | 5′ upstream | T2D | 1432 | 2227 (78) | 637 (22) | 0.422 |
|  |  |  |  |  | NG | 1586 | 2439 (77) | 733 (23) |  |
| rs1417294 | 1qC | G/T | 162767660 | 5′ upstream | T2D | 1376 | 2145 (78) | 607 (22) | 0.644 |
|  |  |  |  |  | NG | 1494 | 2344 (78) | 644 (22) |  |
| rs6701986 |  | G/A | 162768500 | 5′ upstream | T2D | 1397 | 2167 (78) | 627 (22) | 0.473 |
|  |  |  |  |  | NG | 1576 | 2420 (77) | 732 (23) |  |
| rs1578328 |  | C/T | 162769543 | 5′ upstream | T2D | 1408 | 2149 (76) | 667 (24) | 0.414 |
|  |  |  |  |  | NG | 1586 | 2449 (77) | 723 (23) |  |
| rs6660684 |  | A/T | 162770843 | 5′ upstream | T2D | 1402 | 2456 (88) | 348 (12) | 0.747 |
|  |  |  |  |  | NG | 1582 | 2780 (88) | 384 (12) |  |
| rs2211061 |  | G/A | 162771973 | 5′ upstream | T2D | 1402 | 2601 (93) | 203 (7) | 0.254 |
|  |  |  |  |  | NG | 1564 | 2925 (94) | 203 (6) |  |
| rs1338634 |  | G/A | 162772813 | 5′ upstream | T2D | 1423 | 2625 (92) | 221 (8) | 0.218 |
|  |  |  |  |  | NG | 1579 | 2939 (93) | 219 (7) |  |
| rs1338633 |  | G/A | 162773610 | 5′ upstream | T2D | 1400 | 2091 (75) | 709 (25) | 0.886 |
|  |  |  |  |  | NG | 1566 | 2344 (75) | 788 (25) |  |
| rs6698479 |  | A/C | 162774601 | 5′ upstream | T2D | 1394 | 2526 (91) | 262 (9) | 0.136 |
|  |  |  |  |  | NG | 1562 | 2794 (89) | 330 (11) |  |
| rs10918035 |  | A/G | 162776369 | 5′ upstream | T2D | 1391 | 2105 (76) | 677 (24) | 0.262 |
|  |  |  |  |  | NG | 1574 | 2421 (77) | 727 (23) |  |
| rs6697573 |  | G/A | 162779501 | 5′ upstream | T2D | 1416 | 2107 (74) | 725 (26) | 0.995 |
|  |  |  |  |  | NG | 1605 | 2388 (74) | 822 (26) |  |
| rs1338625 |  | A/C | 162780889 | 5′ upstream | T2D | 1420 | 2107 (74) | 733 (26) | **0.039** |
|  |  |  |  |  | NG | 1576 | 2411 (76) | 741 (24) |  |
| rs2153099 |  | G/A | 162782380 | 5′ upstream | T2D | 1398 | 1976 (71) | 820 (29) | 0.054 |
|  |  |  |  |  | NG | 1566 | 2141 (68) | 991 (32) |  |
| rs6662567 |  | C/T | 162783473 | 5′ upstream | T2D | 1433 | 2140 (75) | 726 (25) | **0.040** |
|  |  |  |  |  | NG | 1574 | 2422 (77) | 726 (23) |  |
| rs6679247 |  | G/A | 162786689 | 5′ upstream | T2D | 1427 | 2220 (78) | 634 (22) | 0.490 |
|  |  |  |  |  | NG | 1603 | 2470 (77) | 736 (23) |  |
| rs6426870 |  | T/C | 162790577 | 5′ upstream | T2D | 1396 | 2073 (74) | 719 (26) | **0.014** |
|  |  |  |  |  | NG | 1542 | 2375 (77) | 709 (23) |  |
| rs2275558 (G21S) | 1qC | G/A | 162795744 | Exon 1 | T2D | 1427 | 2193 (77) | 661 (23) | **0.013** |
|  |  |  |  |  | NG | 1626 | 2584 (79) | 684 (21) |  |
| rs2275559 | CNR | G/C | 162796095 | Intron 1 | T2D | 728 | 1252 (86) | 204 (14) | 0.298 |
|  |  |  |  |  | NG | 855 | 1492 (87) | 218 (13) |  |
| rs2275560 | CNR | G/A | 162796290 | Intron 1 | T2D | 727 | 1018 (70) | 436 (30) | 0.647 |
|  |  |  |  |  | NG | 854 | 1183 (69) | 525 (31) |  |
| rs3767374 | CNR | C/T | 162796754 | Intron 1 | T2D | 715 | 1133 (79) | 297 (21) | 0.632 |
|  |  |  |  |  | NG | 850 | 1335 (79) | 365 (21) |  |
| rs4657364 | Reseq | T/C | 162798949 | Intron 1 | T2D | 720 | 1181 (82) | 259 (18) | 0.912 |
|  |  |  |  |  | NG | 838 | 1372 (82) | 304 (18) |  |
| rs1770549 | CNR | T/G | 162862911 | Intron 2 | T2D | 711 | 901 (63) | 521 (37) | 0.172 |
|  |  |  |  |  | NG | 843 | 1028 (61) | 658 (39) |  |
| rs1780337 | CNR | G/T | 162871581 | Intron 2 | T2D | 683 | 787 (58) | 579 (42) | 0.535 |
|  |  |  |  |  | NG | 854 | 1003 (59) | 705 (41) |  |
| rs2792248 | 1qC | A/G | 162891886 | Intron 2 | T2D | 1178 | 1814 (77) | 542 (23) | **0.004** |
|  |  |  |  |  | NG | 1442 | 2121 (74) | 763 (26) |  |
| rs6703114 | CNR | G/T | 162939852 | Intron 2 | T2D | 652 | 954 (73) | 350 (27) | 0.282 |
|  |  |  |  |  | NG | 750 | 1070 (71) | 430 (29) |  |
| rs12089454 | 1qC | T/C | 162951482 | Intron 2 | T2D | 1366 | 1744 (64) | 988 (36) | 0.460 |
|  |  |  |  |  | NG | 1417 | 1836 (65) | 998 (35) |  |
| rs2066087 | CNR | T/C | 162959533 | Intron 2 | T2D | 1371 | 1752 (64) | 990 (36) | 0.562 |
|  |  |  |  |  | NG | 1452 | 1877 (65) | 1027 (35) |  |
| rs1489328 | CNR | T/C | 162959840 | Intron 2 | T2D | 648 | 898 (69) | 398 (31) | 0.119 |
|  |  |  |  |  | NG | 842 | 1211 (72) | 473 (28) |  |
| rs12075411 | 1qC | C/G | 162970593 | Intron 2 | T2D | 1338 | 1796 (67) | 880 (33) | 0.347 |
|  |  |  |  |  | NG | 1232 | 1684 (68) | 780 (32) |  |
| rs7543038 | CNR | G/T | 162990459 | Intron 2 | T2D | 692 | 870 (63) | 514 (37) | 0.156 |
|  |  |  |  |  | NG | 804 | 1051 (65) | 557 (35) |  |
| rs10494422 | Reseq | A/C | 163047639 | Intron 5 | T2D | 698 | 1254 (90) | 142 (10) | 0.790 |
|  |  |  |  |  | NG | 865 | 1559 (90) | 171 (10) |  |
| rs12048214 | Reseq | C/T | 163082091 | Intron 8 | T2D | 702 | 765 (54) | 639 (46) | 0.582 |
|  |  |  |  |  | NG | 840 | 932 (55) | 748 (45) |  |
| rs6426881 | Reseq | C/T | 163083350 | 3′ upstream | T2D | 633 | 1041 (82) | 225 (18) | 0.196 |
|  |  |  |  |  | NG | 848 | 1425 (84) | 271 (16) |  |

^a^ Reseq indicates SNPs identified by resequencing; 1qC, SNPs that showed preliminary evidence of association with T2D (P<0.05) in the French samples of the International Type 2 Diabetes 1q Consortium; CNR, conserved noncoding region; ^b^ SNP alleles are shown as major/minor.

Table S3. *PBX1* SNP genotype counts.

| SNP |  | 1/1 | 1/2 | 2/2 | P |
| --- | --- | --- | --- | --- | --- |
| rs7546193 | T2D | 804 | 526 | 63 | 0.11 |
|  | NG | 948 | 537 | 83 |  |
| rs6672653 | T2D | 1002 | 372 | 32 | 0.90 |
|  | NG | 1154 | 422 | 33 |  |
| rs946253 | T2D | 1000 | 381 | 33 | 0.69 |
|  | NG | 1114 | 426 | 45 |  |
| rs6657417 | T2D | 1123 | 297 | 17 | 0.81 |
|  | NG | 1251 | 312 | 18 |  |
| rs7536427 | T2D | 836 | 456 | 73 | 0.77 |
|  | NG | 941 | 543 | 85 |  |
| rs10918027 | T2D | 802 | 524 | 88 | 0.13 |
|  | NG | 943 | 560 | 77 |  |
| rs10465550 | T2D | 870 | 487 | 75 | 0.71 |
|  | NG | 940 | 559 | 87 |  |
| rs1417294 | T2D | 843 | 459 | 74 | 0.74 |
|  | NG | 933 | 478 | 83 |  |
| rs6701986 | T2D | 843 | 481 | 73 | 0.77 |
|  | NG | 933 | 554 | 89 |  |
| rs1578328 | T2D | 820 | 509 | 79 | 0.67 |
|  | NG | 942 | 565 | 79 |  |
| rs6660684 | T2D | 1080 | 296 | 26 | 0.28 |
|  | NG | 1237 | 306 | 39 |  |
| rs2211061 | T2D | 1208 | 185 | 9 | 0.23 |
|  | NG | 1365 | 195 | 4 |  |
| rs1338634 | T2D | 1210 | 205 | 8 | 0.39 |
|  | NG | 1381 | 207 | 6 |  |
| rs1338633 | T2D | 804 | 483 | 113 | 0.98 |
|  | NG | 901 | 542 | 123 |  |
| rs6698479 | T2D | 1143 | 240 | 11 | 0.33 |
|  | NG | 1258 | 302 | 15 |  |
| rs10918035 | T2D | 796 | 513 | 82 | 0.50 |
|  | NG | 927 | 567 | 80 |  |
| rs6697573 | T2D | 781 | 545 | 90 | 0.76 |
|  | NG | 877 | 634 | 94 |  |
| rs1338625 | T2D | 781 | 545 | 94 | 0.13 |
|  | NG | 923 | 582 | 84 |  |
| rs2153099 | T2D | 686 | 604 | 108 | **0.01** |
|  | NG | 754 | 652 | 174 |  |
| rs6662567 | T2D | 797 | 546 | 90 | 0.14 |
|  | NG | 933 | 573 | 81 |  |
| rs6679247 | T2D | 867 | 486 | 74 | 0.71 |
|  | NG | 951 | 568 | 84 |  |
| rs6426870 | T2D | 771 | 531 | 94 | 0.05 |
|  | NG | 916 | 560 | 79 |  |
| rs2275558 | T2D | 843 | 495 | 80 | 0.09 |
|  | NG | 952 | 486 | 69 |  |
| rs2275559 | T2D | 540 | 172 | 16 | 0.43 |
|  | NG | 649 | 194 | 12 |  |
| rs2275560 | T2D | 346 | 326 | 55 | 0.50 |
|  | NG | 407 | 369 | 78 |  |
| rs3767374 | T2D | 455 | 223 | 37 | 0.90 |
|  | NG | 532 | 271 | 47 |  |
| rs4657364 | T2D | 483 | 215 | 22 | 0.65 |
|  | NG | 566 | 240 | 32 |  |
| rs1770549 | T2D | 285 | 331 | 95 | 0.27 |
|  | NG | 322 | 384 | 137 |  |
| rs1780337 | T2D | 233 | 321 | 129 | 0.79 |
|  | NG | 299 | 405 | 150 |  |
| rs2792248 | T2D | 710 | 394 | 74 | **0.02** |
|  | NG | 799 | 523 | 120 |  |
| rs6703114 | T2D | 347 | 260 | 45 | 0.29 |
|  | NG | 389 | 292 | 69 |  |
| rs12089454 | T2D | 563 | 618 | 185 | 0.63 |
|  | NG | 609 | 618 | 190 |  |
| rs2066087 | T2D | 563 | 626 | 182 | 0.65 |
|  | NG | 619 | 639 | 194 |  |
| rs1489328 | T2D | 310 | 278 | 60 | 0.20 |
|  | NG | 442 | 327 | 73 |  |
| rs12075411 | T2D | 605 | 586 | 147 | 0.43 |
|  | NG | 587 | 510 | 135 |  |
| rs7543038 | T2D | 278 | 314 | 100 | 0.16 |
|  | NG | 361 | 329 | 114 |  |
| rs10494422 | T2D | 565 | 124 | 9 | 0.47 |
|  | NG | 700 | 159 | 6 |  |
| rs12048214 | T2D | 207 | 351 | 144 | 0.45 |
|  | NG | 269 | 394 | 177 |  |
| rs6426881 | T2D | 428 | 185 | 20 | 0.40 |
|  | NG | 601 | 223 | 24 |  |

T2D: type 2 diabetic cases. NG: normoglycemic controls. P values are shown for the chi-squared analysis of the genotype counts.

Table S4. Association analyses of *PBX1* SNPs with quantitative metabolic traits.

|  | BMI (kg/m^2^) | Glucose (mM) | Insulin (pM) | Total Cholesterol (mM) | HDL (mM) | Triglycerides (mM) | APOA1 (g/l) | APOB (g/l) |
| --- | --- | --- | --- | --- | --- | --- | --- | --- |
| n | 1437 | 1400 | 843 | 1318 | 792 | 1311 | 1427 | 1425 |
| Mean | 24.37 ± 0.09 | 5.21 ± 0.02 | 50.01 ± 1.17 | 5.94 ± 0.03 | 1.58 ± 0.01 | 1.12 ± 0.02 | 1.68 ± 0.01 | 1.10 ± 0.01 |
| rs10918027 | 0.195 | 0.270 | 0.274 | 0.279 | 0.808 | 0.065 | 0.383 | 0.465 |
| rs1338625 | 0.224 | 0.242 | 0.284 | 0.747 | 0.928 | 0.150 | 0.421 | 0.751 |
| rs6662567 | 0.473 | 0.087 | 0.311 | 0.641 | 0.898 | 0.096 | 0.409 | 0.766 |
| rs6426870 | 0.258 | 0.203 | 0.204 | 0.935 | 0.957 | 0.240 | 0.338 | 0.694 |
| rs2275558 | 0.317 | 0.644 | 0.378 | 0.982 | 0.637 | 0.196 | 0.667 | 0.649 |
| rs2792248 | 0.665 | 0.736 | 0.311 | 0.665 | 0.556 | 0.475 | 0.952 | 0.465 |

Table S5. Meta-analysis of *PBX1* SNP rs1338625.

| This study | Allele 1 (%) | Allele 2 (%) | P | OR (95%CI) |
| --- | --- | --- | --- | --- |
| T2D | 2107 (74) | 733 (26) | 0.047 | 1.13 (1.00-1.27) |
| NG | 2428 (76) | 750 (24) |  |  |
| Zeggini *et al*. |  |  |  |  |
| T2D | 2874 (75) | 968 (25) | 0.029 | 0.90 (0.82-0.99) |
| NG | 4271 (73) | 1595 (27) |  |  |
| Saxena *et al*. |  |  |  |  |
| T2D | 2207 (78) | 715 (22) | 0.879 | 0.99 (0.87-1.12) |
| NG | 2221 (78) | 625 (22) |  |  |
| Combined Data |  |  |  |  |
| T2D | 7188 (75) | 2416 (25) | 0.618 | 0.98 (0.92-1.05) |
| NG | 8920 (75) | 2970 (25) |  |  |

Table S6. Meta-analysis of *PBX1* SNP rs6426870.

| This study | Allele 1 (%) | Allele 2 (%) | P | OR (95%CI) |
| --- | --- | --- | --- | --- |
| T2D | 2073 (74) | 719 (26) | 0.017 | 1.16 (1.03-1.30) |
| NG | 2392 (77) | 718 (23) |  |  |
| Zeggini *et al*. |  |  |  |  |
| T2D | 2863 (75) | 981 (25) | 0.046 | 0.91 (0.83-1.00) |
| NG | 4266 (73) | 1606 (27) |  |  |
| Saxena *et al*. |  |  |  |  |
| T2D | 2214 (76) | 714 (24) | 0.819 | 0.99 (0.88-1.11) |
| NG | 2211 (75) | 723 (25) |  |  |
| Combined Data |  |  |  |  |
| T2D | 7150 (75) | 2414 (25) | 0.823 | 0.99 (0.93-1.06) |
| NG | 8869 (74) | 3047 (26) |  |  |

Table S7. Meta-analysis of *PBX1* SNP rs4657364.

| This study | Allele 1 (%) | Allele 2 (%) | P | OR (95% CI) |
| --- | --- | --- | --- | --- |
| T2D | 1181 (82) | 259 (18) | 0.912 | 0.99 (0.82-1.19) |
| NG | 1372 (82) | 304 (18) |  |  |
| Zeggini *et al*. [25] |  |  |  |  |
| T2D | 3222 (84) | 620 (16) | 0.749 | 1.02 (0.91-1.14) |
| NG | 4937 (84) | 933 (16) |  |  |
| Saxena *et al*. [24] |  |  |  |  |
| T2D | 2475 (85) | 451 (15) | 0.289 | 1.08 (0.94-1.25) |
| NG | 2509 (86) | 423 (14) |  |  |
| Combined Data |  |  |  |  |
| T2D | 6878 (84) | 1330 (16) | 0.450 | 1.03 (0.95-1.12) |
| NG | 8818 (84) | 1660 (16) |  |  |
